# Hydrogen Sulfide Attenuates Lipopolysaccharide-Induced Inflammation via the P-glycoprotein and NF-κB Pathway in Astrocytes

**DOI:** 10.1007/s11064-022-03840-5

**Published:** 2022-12-08

**Authors:** Yanling Zhao, Han Yan, Xue Liang, Zhenyu Zhang, Xuan Wang, Nianwei Shi, Weihong Bian, Qing Di, He Huang

**Affiliations:** 1 Department of Neurology, The Affiliated Nanjing Brain Hospital of Nanjing Medical University, Nanjing, People’s Republic of China; 2 Department of Geriatrics, Shanghai General Hospital, Shanghai Jiaotong University School of Medicine, Shanghai, People’s Republic of China; 3 Department of Neurology, Tenth People’s Hospital, Tongji University School of Medicine, Shanghai, People’s Republic of China; 4 Research Unit Hypertension and Cardiovascular Epidemiology, KU Louvain Department of Cardiovascular Sciences, University of Leuven, Leuven, Belgium

**Keywords:** Hydrogen sulfide, P-glycoprotein, NF-κB, Astrocyte, Neuroinflammation

## Abstract

Astrocyte activation is key in neurodegenerative diseases. Hydrogen sulfide (H_2_S) exhibits neuroprotective effects on astrocytes, although the underlying molecular mechanism remains unclear. Here, we explored the effects of H_2_S on lipopolysaccharide (LPS)-induced astrocyte activation and astrocyte-mediated neuroinflammation. After inducing primary astrocytes via LPS exposure, H_2_S levels were altered. The generation and secretion of inflammatory mediators by astrocytes and their interrelation with P-glycoprotein (P-gp), an important transporter belonging to the ABC transporter family, were assessed. Activated astrocytes showed upregulated glial fibrillary acidic protein (*GFAP*) mRNA expression, and significantly increased proinflammatory factor mRNA/protein expression and release. The secretory capacity of astrocytes was reduced, with significantly decreased proinflammatory factor levels in culture supernatant after P-gp inhibitor verapamil pretreatment. The increase in the intracellular H_2_S level inhibited LPS-induced GFAP expression and P65 nuclear entry in astrocytes. mRNA expression and release of proinflammatory factors were reduced significantly, with no significant changes in cytoplasmic protein expression. S-sulfhydration levels increased significantly with the increased concentration of sodium hydrosulfide or S-adenosyl-l-methionine addition, with only moderate changes in astrocyte P-gp expression. H_2_S regulates NF-κB activation, leads to S-sulfhydration of P-gp, and inhibits the biosynthesis and secretion of proinflammatory factors by astrocytes. The regulatory effects of H_2_S on astrocytes may have clinical value for exploring new therapeutic strategies against neurodegenerative diseases.

**Supplementary Information:**

The online version contains supplementary material available at 10.1007/s11064-022-03840-5.

## Introduction

Neuroinflammatory reactive astrocytes are widely present in neurodegenerative diseases. They mediate inflammatory and pathological processes in Alzheimer's disease (AD), multiple sclerosis (MS), and amyotrophic lateral sclerosis [1, 2]. Following exposure to inflammatory stimuli (e.g., lipopolysaccharides [LPS]) or under pathological conditions, the activated astrocytes not only lose their normal physiological functions but also secrete various toxins (e.g., inflammatory factors), which perpetuate the inflammatory response and trigger a vicious circle, disrupting the homeostasis of the nervous system microenvironment and inducing the death of surrounding neurons and oligodendrocytes [3]. The obstruction of the biosynthesis and secretion of inflammatory factors by activated astrocytes is an important strategy to maintain neuronal microenvironment homeostasis and treat neurodegenerative diseases [4, 5].

P-glycoprotein (P-gp) is an important transporter belonging to the ABC transporter family and is expressed in vascular endothelial cells, astrocytes, and microglia in the central nervous system (CNS) [6–8]. Recent studies suggested that P-gp also exerts immunomodulatory activity and is a potential immunotherapeutic target [9, 10]. Previous data showed that in addition to exogenous substances, the substrates of P-gp also include various endogenous molecules, e.g., inflammatory factors, hormones, and β-amyloid peptides [11–13]. The functional changes of P-gp in patients with neurological diseases (e.g., epilepsy, MS, and AD) are accompanied by enhanced inflammatory responses [14–16]. Therefore, P-gp dysfunction contributes to the inflammatory process in CNS-related diseases.

Hydrogen sulfide (H_2_S) is an important gaseous signaling molecule in the human body. It is mainly produced from l-cysteine (Cys) and homocysteine (Hcy) via the enzymatic action of cystathionine β-synthase (CBS), cystathionine γ-lyase (CSE), and 3-mercaptopyruvate sulfotransferase (3MST) [17]. Astrocytes express high levels of CBS and are the main source of H_2_S in the brain [18]. H_2_S exhibits not only antioxidant and antiapoptotic effects but also anti-inflammatory properties [17]. H_2_S levels in the brain decrease with age. It was previously found that H_2_S levels and CBS activity in astrocytes decrease significantly in patients with neurological diseases [e.g., AD, Parkinson’s disease (PD), and MS], while the restoration of H_2_S levels or the enhancement of CBS activity in the brain exerts neuroprotective effects by alleviating neuroinflammation [18, 19]. Hence, H_2_S exhibits anti-neuroinflammatory effects by acting on astrocytes, but the underlying molecular mechanism remains unclear.

Gazzano et al. uncovered that combining H_2_S and doxorubicin yields a significantly greater efficacy than monotherapy with doxorubicin alone against osteosarcoma with a high P-gp expression because H_2_S reduces the efflux of doxorubicin from osteosarcoma cells by inhibiting P-gp, thereby enhancing its therapeutic efficacy [20]. In other words, H_2_S can alter transportation across the cell membrane by modulating the activity of P-gp on the membrane of osteosarcoma cells. The question remains whether H_2_S also affects the efflux of other toxic substances (e.g., inflammatory factors) from activated astrocytes to regulate inflammatory responses in the neuronal microenvironment. We hypothesize that H_2_S may regulate neuroinflammatory responses by affecting P-gp activity in astrocytes. Here, we explored the effects of H_2_S on LPS-induced astrocyte activation and astrocyte activation-mediated neuroinflammation, as well as the mechanisms underlying these effects.

## Materials and Methods

All reagents were purchased from Sinopharm Chemical Reagents (Shanghai, China) unless indicated otherwise.

### Cell Isolation, Culture, and Treatment

One to 3 days old neonatal Sprague Dawley (SD) rats were obtained from the Laboratory Animal Resources, Chinese Academy of Sciences (Shanghai, China). All experimental procedures and protocols strictly conformed to the recommended National Institutes of Health Guidelines for the Care and Use of Laboratory Animals and were approved by the Animal Experimentation Ethics Committee of Tenth People's Hospital Affiliated with Tongji University. Neonatal rats were euthanized by immersion in 75% ethanol and then transferred onto an ultraclean workbench to harvest their brains and isolate the cerebral cortical tissues. The tissues were minced into 1 mm^3^ pieces, which were then immersed in phosphate-buffered saline (PBS) containing 1% penicillin/streptomycin and trypsinized with 0.25% trypsin at 37 °C for 10 min to prepare the cell suspension. After filtering through 100, 200, and 400 μm pore mesh, the cell suspension was centrifuged at 300×*g* for 5 min. The cells were resuspended in the complete astrocyte medium (CM-R137; Procell, Wuhan, China) and plated in flasks precoated with polylysine, then cultured at 37 °C in a 95% air/5% CO_2_ incubator. After being cultivated for 48 h, the medium was replaced for the first time, and the cell culture was subsequently continued with the medium being changed every 3 days. Finally, immunostaining of glial fibrillary acidic protein (GFAP) (SC-58766; Santa Cruz, California, USA), DAPI counterstaining, and cell counts of GFAP-positive cells relative to total DAPI-positive cells were performed to guarantee the percentage of cultured pure primary astrocytes was above 95%.

### Cell Counting Kit-8 (CCK-8) Assay

After astrocytes were processed, cell viability was assessed using a CCK-8 assay kit according to the manufacturer’s instructions (C0037; Beyotime Biotechnology, Shanghai, China). The absorbance values at 450 nm (OD450) were measured as per the instructions provided with the kit. Cell viability was determined as follows: Optical density (OD) of the experimental group/OD of the control group × 100%.

### Enzyme-Linked Immunosorbent Assay (ELISA)

For ELISA, cell culture supernatants (1 mL) of astrocytes were used. Interleukin (IL)-1β, IL-6, and tumor necrosis factor (TNF)-α content in the astrocyte medium were measured using the relevant ELISA kits (ab100768, ab234570, and ab100785; Abcam, Cambridge, UK), according to the manufacturer's instructions.

### Western Blotting

Western blotting was performed as previously reported [21]. Cells were homogenized with a lysis buffer containing radioimmunoprecipitation assay buffer (Merck Millipore, Billerica, MA, USA) and protease inhibitor cocktails (1:1000; Sigma-Aldrich, St. Louis, MO, USA) on ice before centrifugation (10,000×*g* for 10 min) to remove insoluble material. Protein lysate (15 µg) was subjected to 8 or 12% sodium dodecyl sulfate–polyacrylamide gel electrophoresis (SDS-PAGE), followed by transfer to a nitrocellulose membrane (Merck Millipore), which was blocked in 5% bovine serum albumin (BSA)/PBS for 1 h before an overnight incubation at 4 °C with primary antibodies (rabbit polyclonal anti-IL-1β, -IL-6, and -TNF-α [1:1000; ab254360, ab259341, and ab183218; Abcam], as well as anti-P65 [1:1000; ab76302] and -P-gp [1:2000; ab170904]) diluted in 5% BSA/PBS. Protein expression was detected via enhanced chemiluminescence (Merck Millipore) and normalized against Tubulin.

According to the manufacturer’s instructions, cytoplasmic and nuclear proteins were isolated from astrocytes using the Nuclear and Cytoplasmic Protein Extraction Kit (P0028; Beyotime Biotechnology). Histone H3 was used as an internal reference for the detection of nuclear P65.

### RNA Extraction, Reverse Transcription, and Real-Time PCR (RT-PCR)

Total RNA was extracted from astrocytes using TRIzol reagent (15596026; Ambion, Foster City, CA, USA) according to the manufacturer’s instructions. Extracted RNA (1 µg) was used for first-strand cDNA synthesis with a PrimeScript RT reagent kit (RR037A; Takara, Shiga, Japan) according to the manufacturer’s instructions. RT-PCR analyses were performed using a LightCycler 96 (Roche, Basel, Switzerland) with SYBR Premix Ex TaqII (Tli RNaseH Plus, RR420A; Takara). Following initial denaturation at 95 °C for 3 min, PCR was continued for 40–45 cycles at 95 °C for 10 s and 60 °C for 60 s. Relative gene expression was calculated using the 2^−ΔΔCt^ method and normalized against the housekeeping gene *actb* to compensate for variations in input cDNA [21]. The PCR primers used in this study are listed in Table 1.

**Table 1:** PCR primers used in this study

Primer name	Primer sequence (5′ → 3′)
P-gp Forward	CTTCGACCCACACTTCAGCTA
P-gp Reverse	AGCCGGTACCCGCAATG
TNF-α Forward	ATGGGCTCCCTCTCATCAGT
TNF-α Reverse	GCTTGGTGGTTTGCTACGAC
IL-1β Forward	TACCTATGTCTTGCCCGTGGAG
IL-1β Reverse	ATCATCCCACGAGTCACAGAGG
IL-6 Forward	TCCTACCCCAACTTCCAATGCTC
IL-6 Reverse	TTGGATGGTCTTGGTCCTTAGCC
GFAP Forward	CAAGAAACAGAAGAGTGGTATCGGT
GFAP Reverse	ACTCAAGGTCGCAGGTCAAGG
Actin Forward	ATGGATGACGATATCGCTGCG
Actin Reverse	GGTGACAATGCCGTGTTCAAT

### Rhodamine 123 Accumulation Assay

After drug treatment, cells were washed with PBS. After a 90 min incubation with Rhodamine 123 (2 μM) (C2007; Beyotime Biotechnology) at 37 ℃, Rhodamine 123 was washed with cold PBS and 1% Triton X-100, and the cells were kept on ice until analysis. After being incubated with the DAPI staining solution for 10 min, the accumulation of Rhodamine 123 was measured using a Leica fluorescence microscope (Leica Microsystems GmbH, Germany). The stronger the efflux activity of P-gp in cells, the weaker the fluorescence intensity of intracellular Rhodamine 123. The mean fluorescence of Rhodamine 123 in cells was calculated using ImageJ 1.46 software.

### Modified Biotin Switch (S-sulfhydration) Assay

The assay was performed as described previously [22], with modifications. Briefly, cells were homogenized in HEN buffer [250 mM Hepes–NaOH (pH 7.7), 1 mM EDTA, and 0.1 mM neocuproine] supplemented with 100 μM deferoxamine and centrifuged at 13,000 × g for 30 min at 4 °C. Cell lysates (240 µg) or pure Tubulin (0.3 µg) were added to the blocking buffer (HEN buffer adjusted to 2.5% SDS and 20 mM methyl methanethiosulfonate [MMTS]) at 50 ℃ for 20 min with frequent vortexing. MMTS was then removed with acetone, and the proteins were precipitated at − 20 ℃ for 20 min. After acetone removal, the proteins were resuspended in HENS buffer (HEN buffer adjusted to 1% SDS). Four millimolars of biotin-HPDP (21,341; ThermoFisher Scientific, USA) in dimethyl sulfoxide was added to the solution without ascorbic acid. After incubation for 3 h at 25 ℃, biotinylated proteins were precipitated using streptavidin-agarose beads, which were then washed with HENS buffer. The biotinylated proteins were eluted using an SDS-PAGE sample buffer and subjected to western blotting. For quantitation of protein sulfhydration, samples were run on blots alongside total lysates ("loads") and subjected to immunoblotting with antibodies specific to each protein.

### Statistical Analysis

Western blotting results were analyzed and processed using the Image Pro-Plus 6.0 software. All data analysis and statistical analysis were conducted using the SPSS 22.0 statistical software. All data were expressed as the mean ± standard deviation ($\overline{x}$ ± SD). The pairwise comparison between different groups was performed using the least significant difference function of one-way ANOVA. p < 0.05 indicates the presence of a statistically significant difference.

## Results

### LPS Induced the Inflammatory Activation of Astrocytes and Increased the Secretion of Inflammatory Cytokines

**Fig. 1:**
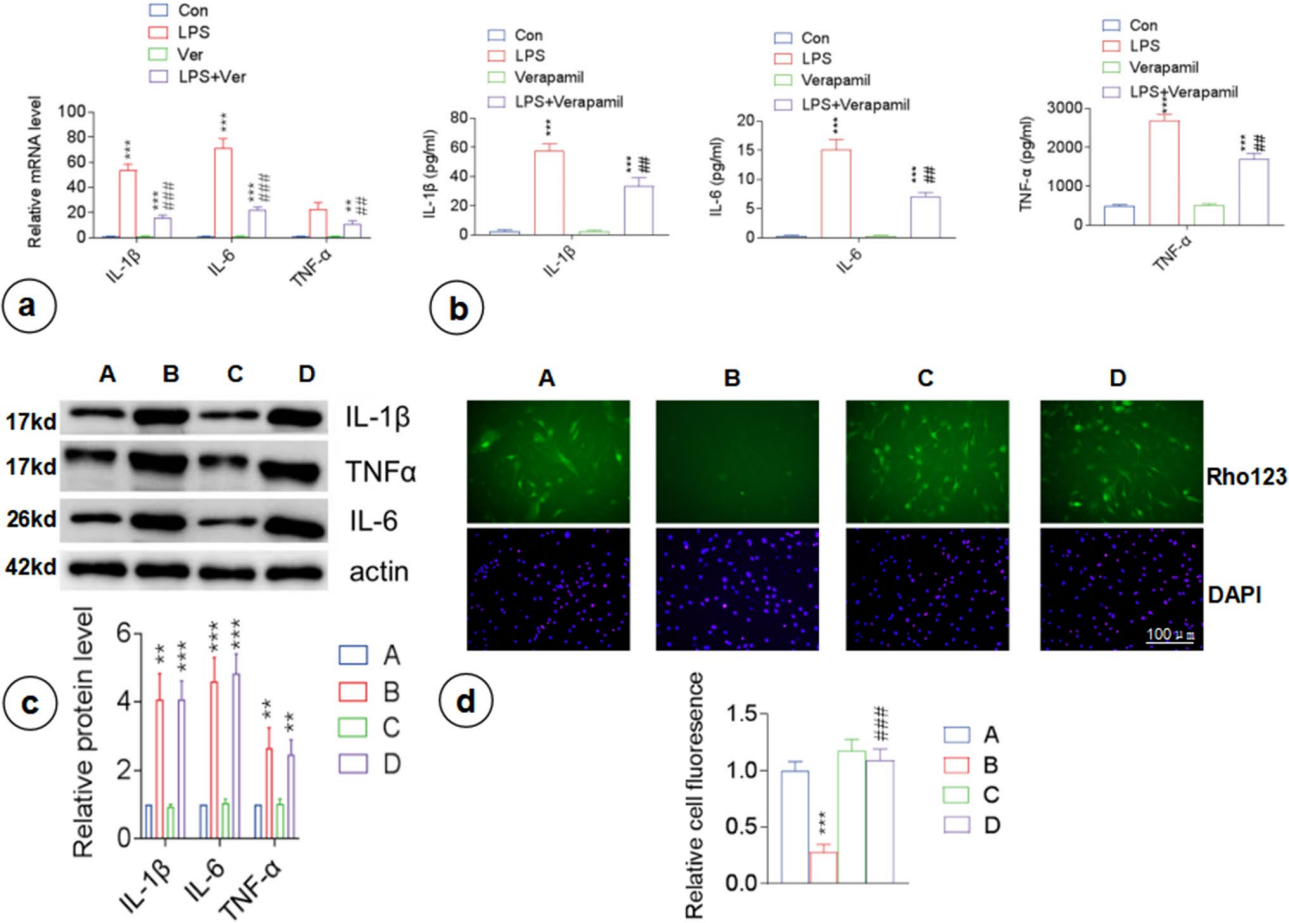
P-gp regulates the extracellular secretion of proinflammatory factors in primary astrocytes stimulated by LPS. LPS-stimulated proinflammatory cytokine (IL-1β, IL-6, and TNF-α) expression and extracellular secretion in primary astrocytes were suppressed following administration of the P-gp inhibitor verapamil (70 μM). **a** RT-PCR analysis of mRNA expression of proinflammatory factors in astrocytes.** b** ELISA to detect proinflammatory factor levels in the cell culture supernatant. **c** Western blotting to detect proinflammatory factor levels in the cytoplasm. **d** Rhodamine 123 (Rho123) accumulation assay to detect P-gp function: green fluorescence for intracellular Rhodamine 123, blue fluorescence for nucleus. A, Con; B, LPS; C, verapamil; D, LPS + verapamil. n = 3. **p < 0.01 vs Con, ***p < 0.001 vs Con; ##p < 0.01 vs LPS, ###p < 0.001 vs LPS

After being treated with 1 μg/mL of LPS (Aladdin, Shanghai, China) for 48 h, GFAP expression in primary astrocytes (Online Resource 1; Fig. S1) was significantly upregulated (p < 0.01) (Online Resource 1; Fig. S2), while its proliferative capacity remained unaffected (Online Resource 1; Fig. S3a). LPS exposure significantly increased the expression levels of intracellular proinflammatory factors (IL-1β, IL-6, and TNF-α) (p < 0.001) (Fig. 1a), as well as that of the extracellular inflammatory factors in the culture supernatant (p < 0.001) (Fig. 1b), compared with the control group. In other words, LPS induced the inflammatory activation of astrocytes and increased the secretion of inflammatory factors from astrocytes.

### P-gp Participated in the LPS-Induced Secretion of Inflammatory Factors from Astrocytes

Primary rat astrocytes were pretreated with 70 μM of verapamil (MedChemExpress, USA) (P-gp inhibitor) for 48 h [23]. Blank control, verapamil, LPS, and LPS + verapamil groups were included. As shown in Fig. 1, there were no significant differences in the intracellular and extracellular levels of inflammatory factors between the verapamil group and the baseline control group (p > 0.05). Both LPS and LPS + verapamil groups had significantly higher expression of intracellular inflammatory factors (IL-1β, IL-6, and TNF-α) than the control group (Fig. 1c). Besides, there was no significant difference in the expression of intracellular inflammatory factors between the two groups (p > 0.05) (Fig. 1c). However, the LPS + verapamil group displayed significantly lower mRNA expression levels and extracellular (in culture supernatant) inflammatory factor levels than the LPS group (Fig. 1a, b).

Meanwhile, the evaluation of P-gp transport via Rhodamine 123 accumulation assay (Fig. 1d) showed that the intracellular accumulation of Rhodamine 123 in the LPS group was significantly lower than that in the baseline control group (p < 0.001) but increased significantly following verapamil treatment in the LPS + verapamil group (p < 0.001), i.e., LPS-induced transportation across cell membrane had been suppressed. Therefore, verapamil could reduce the LPS-induced secretion of inflammatory factors by inhibiting the transport function of P-gp.

### Effects of Exogenous H_2_S on the LPS-Induced Inflammatory Responses of Astrocytes

#### Sodium Hydrosulfide (NaHS) Inhibited the Inflammatory Activation of Astrocytes

**Fig. 2:**
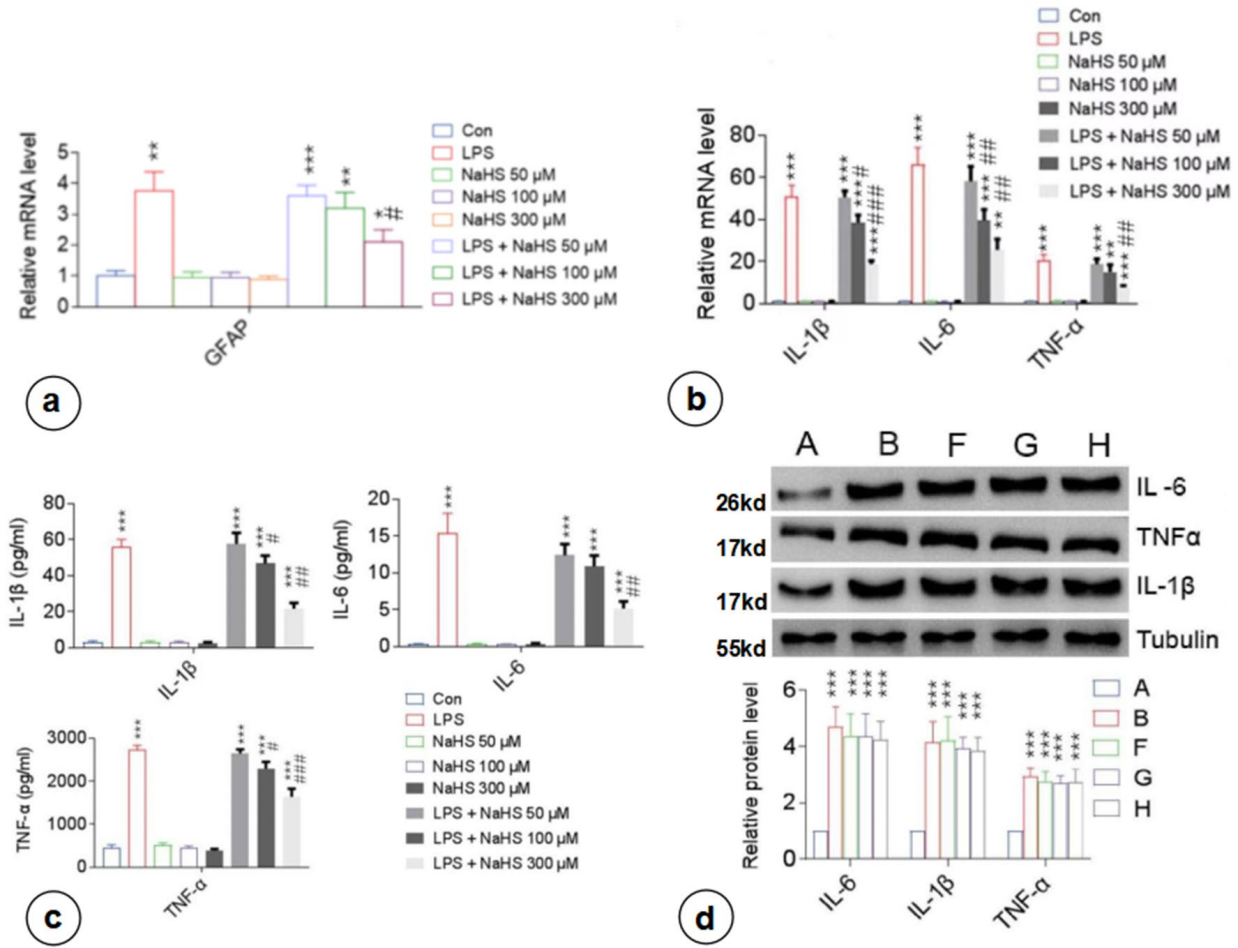
Exogenous H_2_S supplementation inhibited the LPS-induced production and secretion of inflammatory factors by primary astrocytes. LPS-stimulated GFAP mRNA expression, proinflammatory cytokine (IL-1β, IL-6, and TNF-α) expression and extracellular secretion in astrocytes were suppressed following administration of NaHS (50, 100, and 300 µM). **a** RT-PCR of GFAP mRNA expression in primary astrocytes. **b** RT-PCR analysis of proinflammatory factor mRNA expression in primary astrocytes. **c** ELISA of proinflammatory factor levels in the cell culture supernatant.** d** Western blotting analysis of cytoplasmic proinflammatory factor levels. A, Con; B, LPS; F, LPS + NaHS 50 µM; G, LPS + NaHS 100 µM; H, LPS + NaHS 300 µM. n = 3. **p < 0.01 vs Con, ***p < 0.001 vs Con; ##p < 0.01 vs LPS, ###p < 0.001 vs LPS

Three concentrations of NaHS (H_2_S donor; 50, 100, and 300 µM) were selected based on the maximum physiologically tolerated concentration of H_2_S [24] to assess its effects on the inflammatory activation of astrocytes (Online Resource 1; Fig. S3b). The comparative analysis between the control, LPS, NaHS (50, 100, and 300 µM), and LPS + NaHS (LPS + NaHS 50 μM, LPS + NaHS 100 μM, and LPS + NaHS 300 µM) groups showed that NaHS did not affect the baseline mRNA expression of GFAP compared with the control group (Fig. 2a). *GFAP* mRNA expression in the LPS group increased significantly and was higher than that in the LPS + NaHS group, in which GFAP mRNA expression gradually decreased with the increasing NaHS concentration, suggesting that NaHS inhibited the LPS-induced inflammatory activation of astrocytes.

#### NaHS Inhibited the LPS-Induced Production and Secretion of Inflammatory Factors by Astrocytes

Compared with the control group, NaHS treatment alone did not affect the mRNA expression of inflammatory factors (*Il1b*, *Il6*, and *tnfa*) in astrocytes (Fig. 2b). The LPS group showed significantly upregulated mRNA expression of inflammatory factors, which were suppressed upon pretreatment with NaHS in the LPS + NaHS group. Besides, the expression levels of inflammatory factors decreased gradually with the increasing NaHS concentration.

Our analysis also revealed that there were no significant changes in the levels of inflammatory factors in the culture supernatant between the NaHS group and the baseline control group (p > 0.05) (Fig. 2c). The levels of all inflammatory factors in the culture supernatant of the LPS + NaHS group cells were lower than those of the LPS group cells and were negatively correlated with the concentration of NaHS. However, it is interesting to note that there were no significant differences in the intracellular expression of inflammatory factors between LPS + NaHS and LPS groups, regardless of the concentration of NaHS (p > 0.05) (Fig. 2d), i.e., NaHS inhibited LPS-induced secretion of inflammatory factors from astrocytes.

### NaHS Inhibited LPS-Induced NF-kB Activation in Astrocytes

**Fig. 3:**
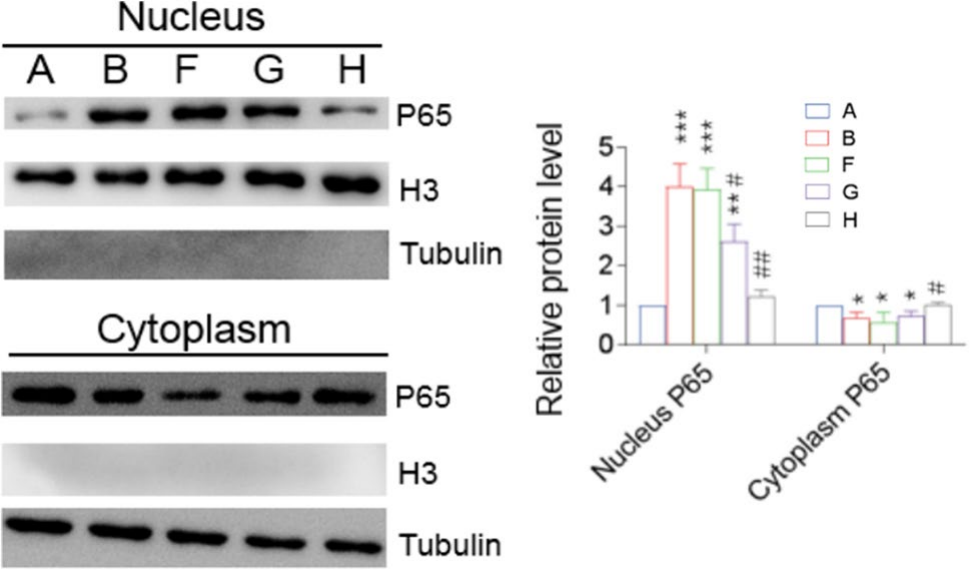
NaHS inhibited the LPS-induced activation of the NF-kB pathway in astrocytes. The nuclear entry of LPS-stimulated P65 was inhibited by NaHS, and the inhibition increased with increasing NaHS concentration. The distribution of P65 in the nucleus and cytoplasm of astrocytes was detected via western blotting. A, Con; B, LPS; F, LPS + NaHS 50 µM; G, LPS + NaHS 100 µM; H, LPS + NaHS 300 µM. n = 3. *p < 0.05 vs Con, **p < 0.01 vs Con, ***p < 0.001 vs Con; #p < 0.05 vs LPS, ##p < 0.01 vs LPS

LPS group had a higher nuclear P65 expression (p < 0.001), reflecting NF-kB pathway activation [25], and a lower cytoplasmic P65 expression (p < 0.05) than the control group (Fig. 3). Compared with the LPS group, the nuclear P65 expression in the LPS + NaHS group reduced gradually, whereas the cytoplasmic P65 expression increased gradually with the increasing concentration of NaHS, suggesting that NaHS inhibited the nuclear entry of P65 and downregulated the expression of inflammatory factors.

### NaHS Regulated the Secretion of Inflammatory Factors from Astrocytes by Inhibiting the Transport of P-gp via S-sulfhydration

**Fig. 4:**
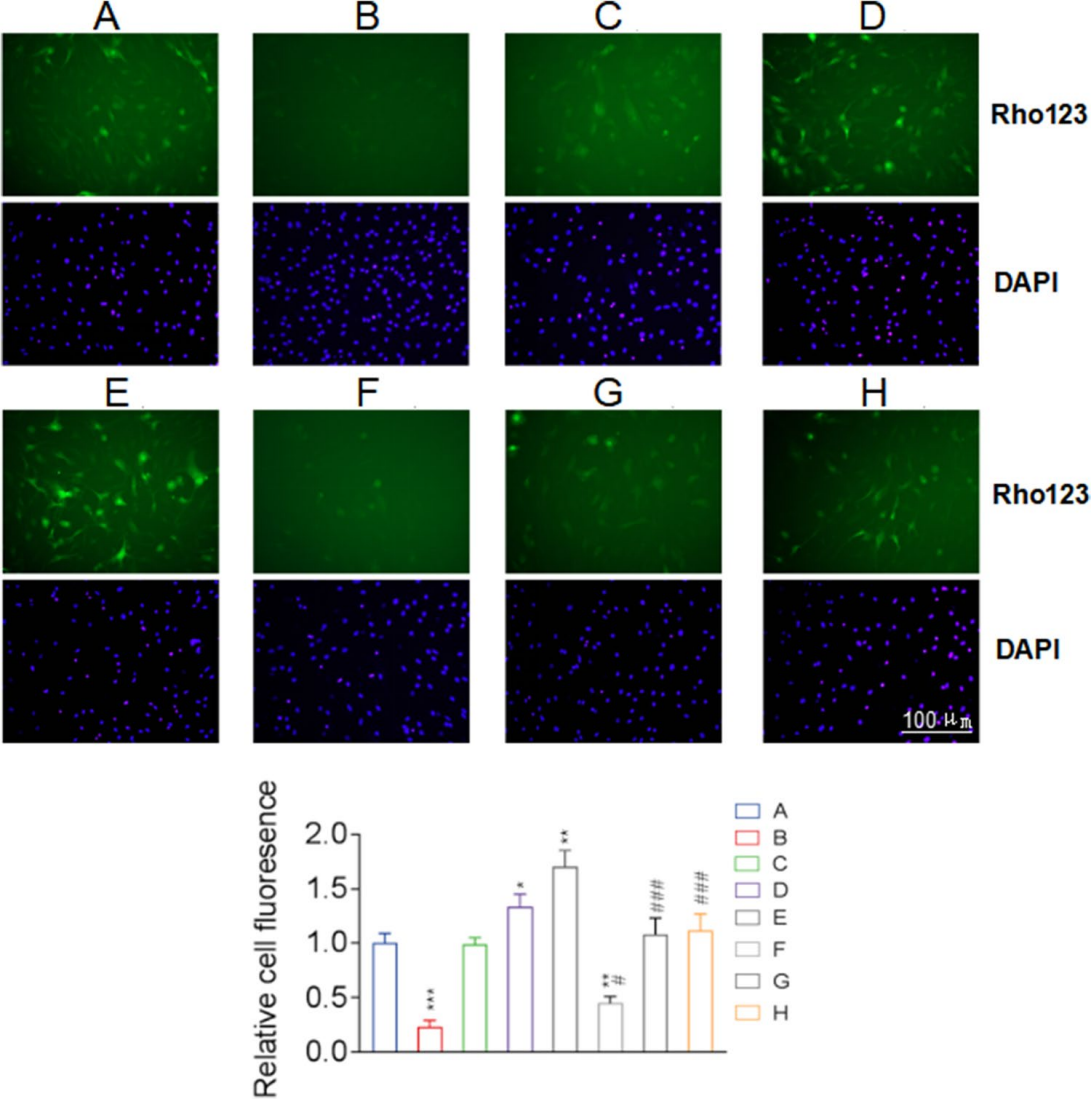
NaHS regulates astrocyte secretory activity through inhibiting P-gp-mediated transport. The enhanced transport of P-gp in LPS-stimulated astrocytes was inhibited by NaHS, as shown by the accumulation of Rhodamine 123 (Rho123) in astrocytes, which was inversely proportional to the concentration of NaHS. Green fluorescence for intracellular Rhodamine 123, and blue fluorescence for the nucleus. A, Con; B, LPS; C, NaHS 50 µM; D, NaHS 100 µM; E, NaHS 300 µM; F, LPS + NaHS 50 µM; G, LPS + NaHS 100 µM; H, LPS + NaHS 300 µM. n = 3. *p < 0.05 vs Con, **p < 0.01 vs Con, ***p < 0.001 vs Con; #p < 0.05 vs LPS, ###p < 0.001 vs LPS

The assessment of the effects of NaHS on P-gp-mediated secretion via Rhodamine 123 accumulation assay showed that the intracellular accumulation of Rhodamine 123 in the LPS + NaHS group was significantly higher than that in the LPS group and increased with the increasing concentration of NaHS, indicating that NaHS inhibited the LPS-enhanced P-gp function (Fig. 4). NaHS could enhance the intracellular accumulation of Rhodamine 123 in astrocytes (NaHS group) even under baseline conditions (without LPS-induced inflammatory activation).

**Fig. 5:**
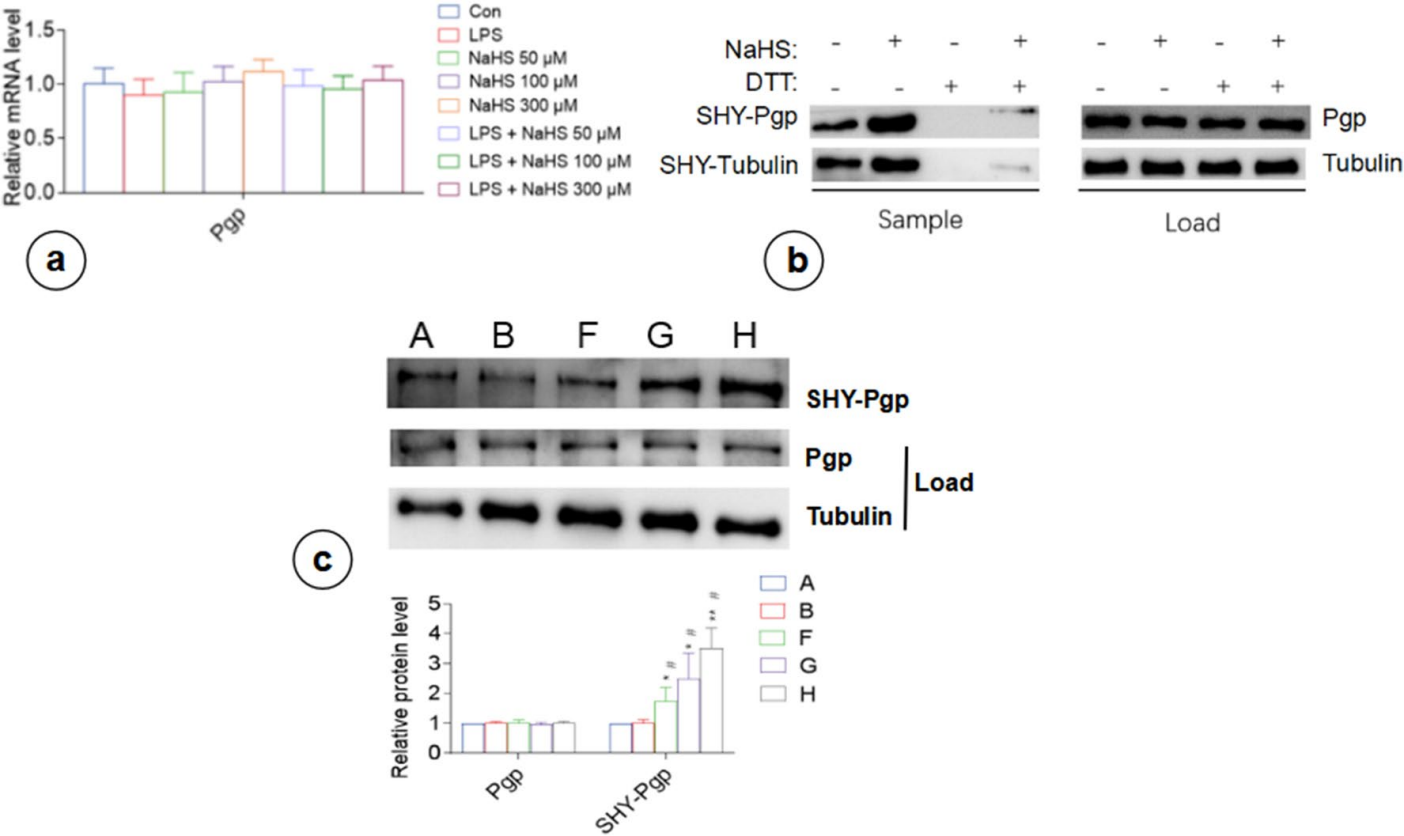
Exogenous H_2_S supplementation increases astrocyte P-gp sulfation without affecting its expression at the mRNA and protein level. **a** RT-PCR analysis of astrocytic P-gp-encoding gene expression. **b** P-gp was S-sulfhydrated by NaHS (300 µM), detected via modified biotin switch (S-sulfhydration) assay. **c** Biotin switch (S-sulfhydration) analysis to detect the level of P-gp sulfation in astrocytes. A, Con; B, LPS; F, LPS + NaHS 50 µM; G, LPS + NaHS 100 µM; H, LPS + NaHS 300 µM. n = 3. DTT, DL-Dithiothreitol; SHY-Pgp, sulfhydrated Pgp; SHY-Tubulin, sulfhydrated Tubulin. *p < 0.05 vs Con, **p < 0.01 vs Con; #p < 0.05 vs LPS

Subsequent analysis of P-gp expression at mRNA and protein levels in astrocytes under different treatment conditions uncovered that P-gp expression at both mRNA and protein levels in H_2_S, LPS, and LPS + NaHS groups did not differ significantly from that in the control group (p > 0.05) (Fig. 5a, c). S-sulfhydration assay showed that LPS and control groups had lower levels of S-sulfhydrated P-gp compared to the LPS + NaHS group, which was positively correlated with the concentration of NaHS (Fig. 5b, c). Therefore, NaHS regulated the S-sulfhydration of P-gp to altered its efflux activity, eventually affecting the secretion of inflammatory factors from astrocytes.

### Effects of Endogenous H_2_S Levels on LPS-Induced Inflammatory Responses of Astrocytes

The endogenous level of H_2_S was altered using the activator and inhibitor of CBS, i.e., S-adenosyl-l-methionine (SAM, Sigma-Aldrich, 0.1 mmol/L) and amino-oxyacetic acid (AOAA, Sigma-Aldrich, 1 mmol/L), respectively, to further examine its effects on the LPS-induced inflammatory response (Online Resource 1; Fig. S3c) [26]. The experiment was designed to include control, LPS, SAM, AOAA, LPS + SAM, LPS + AOAA, and LPS + SAM + AOAA groups for different treatment conditions.

#### Effects of CBS on the LPS-Induced Biosynthesis and Secretion of Inflammatory Factors by Astrocytes

**Fig. 6:**
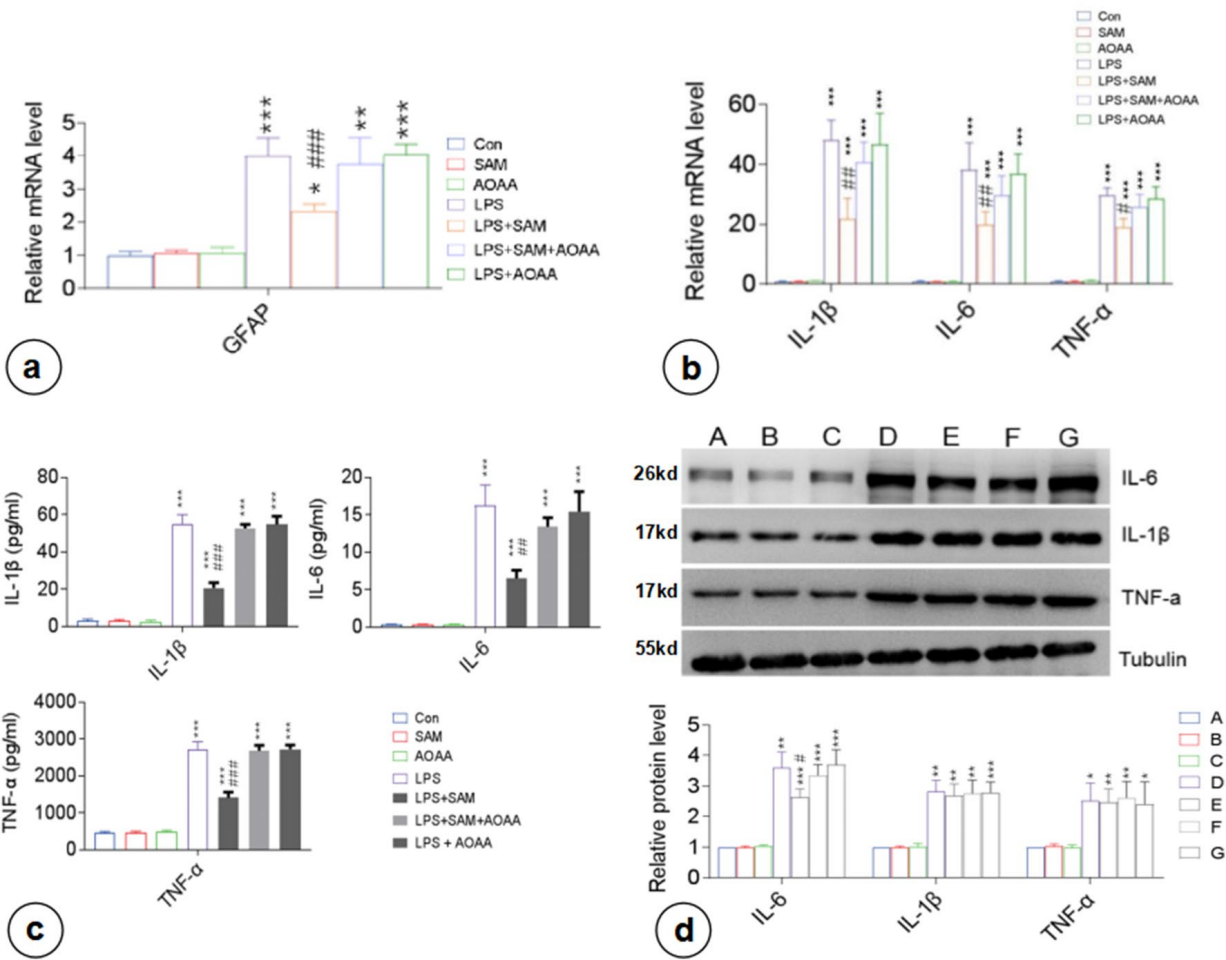
Effects of endogenous H_2_S on the inflammatory activation of astrocytes and the LPS-induced production and secretion of inflammatory factors by astrocytes. Upon LPS stimulation, GFAP mRNA expression and proinflammatory cytokine (IL-1β, IL-6, and TNF-α) expression and secretion in primary astrocytes were suppressed following administration of SAM, which could be eliminated by the addition of the CBS inhibitor AOAA. **a** RT-PCR of GFAP mRNA expression in primary astrocytes. **b** RT-PCR of proinflammatory factor expression in primary astrocytes. **c** ELISA of proinflammatory factor levels in the cell culture supernatant. **d** Western blotting analysis of cytoplasmic proinflammatory factor levels. A, Con; B, SAM; C, AOAA; D, LPS; E, LPS + SAM; F, LPS + SAM + AOAA; G, LPS + AOAA. n = 3. **p < 0.01 vs Con, ***p < 0.001 vs Con; #p < 0.05 vs LPS, ##p < 0.05 vs LPS, ###p < 0.001 vs LPS

SAM and AOAA groups did not differ significantly from the control group in terms of *GFAP* mRNA expression in astrocytes, as well as the expression levels of inflammatory factors at mRNA and protein levels (intracellular and extracellular) (p > 0.05) (Fig. 6). LPS + SAM group astrocytes displayed significantly lower mRNA expression of *GFAP* and inflammatory factors (*Il1b*, *Il6*, and *Tnfɑ*), as well as extracellular levels of inflammatory factors in the culture supernatant compared to the LPS group (Fig. 6a–c). Besides, the LPS + SAM group also had a reduced intracellular expression of IL-6 (p < 0.05) (Fig. 6d). However, there were no significant differences in the intracellular expression of IL-1β and TNF-α between the two groups (p > 0.05) (Fig. 6d). Further analysis revealed that such regulatory effects of SAM could be suppressed by AOAA, whereby the mRNA expression of GFAP and inflammatory factors, as well as the intracellular expression and extracellular levels of inflammatory factors in LPS + SAM + AOAA and LPS + AOAA groups, did not differ significantly from those in the LPS group (Fig. 6a–c). The results suggested that the increased CBS activity could inhibit the LPS-induced inflammatory activation of astrocytes by downregulating the expression and secretion of inflammatory factors, especially TNF-α and IL-1β.

#### Effects of CBS on LPS-Induced NF-kB Activation in Astrocytes

**Fig. 7:**
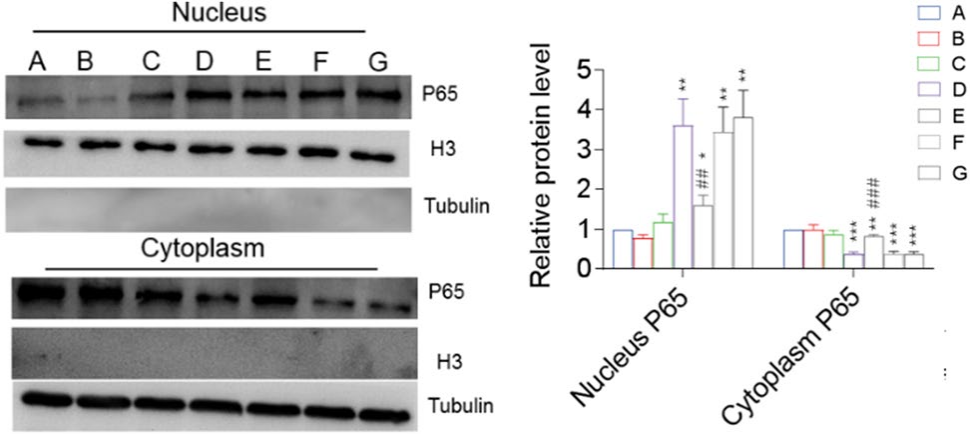
Effects of endogenous H_2_S on the NF-kB pathway of LPS-induced astrocytes. The nuclear entry of LPS-stimulated P65 was inhibited by SAM, which could be inhibited by the addition of AOAA. The distribution of P65 in the nucleus and cytoplasm of astrocytes was detected by western blotting. A, Con; B, SAM; C, AOAA; D, LPS; E, LPS + SAM; F, LPS + SAM + AOAA; G, LPS + AOAA. n = 3. *p < 0.01 vs Con, **p < 0.01 vs Con, ***p < 0.001 vs Con; ##p < 0.05 vs LPS, ###p < 0.001 vs LPS

The nuclear and cytoplasmic distributions of P65 did not differ in SAM and AOAA groups compared with the control group, while the LPS, LPS + SAM + AOAA, and LPS + AOAA groups showed increased nuclear localization (p < 0.01) and decreased cytoplasmic localization of P65 (p < 0.001) (Fig. 7). The nuclear expression of P65 in the LPS + SAM group was higher than that in the control group (p < 0.05), but significantly lower than that in the LPS, LPS + SAM + AOAA, and LPS + AOAA groups (LPS + SAM vs LPS, p < 0.01), while the opposite was true for the cytoplasmic expression of P65 (LPS + SAM vs LPS, p < 0.001) (Fig. 7). The results showed that the increased level of endogenous H_2_S downregulated the expression of inflammatory factors by reducing the LPS-induced nuclear entry of P65 and inhibiting the activation of the NF-κB pathway in astrocytes.

### CBS Inhibited the P-gp-Mediated Transport in Astrocytes by Regulating S-Sulfhydration

**Fig. 8:**
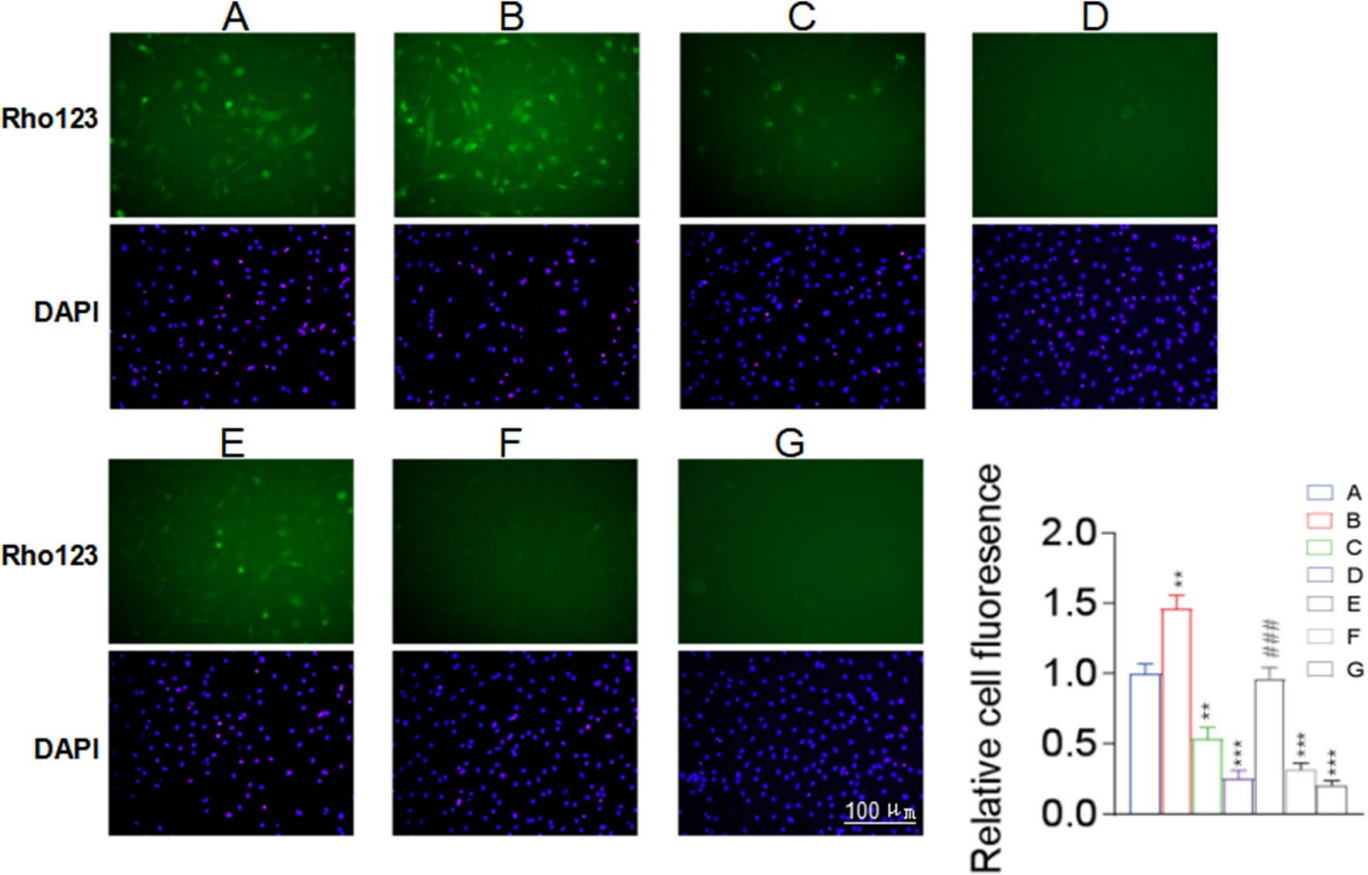
The effect of endogenous H_2_S on the function of P-gp in astrocytes. SAM inhibited basal and LPS-activated P-gp transport activity in astrocytes, while AOAA reversed or enhanced P-gp transport activity in astrocytes. Green fluorescence for intracellular Rhodamine 123, and blue fluorescence for the nucleus. Rhodamine 123 (Rho123) accumulation assay was used to detect the transport function of P-gp in astrocytes. A, Con; B, SAM; C, AOAA; D, LPS; E, LPS + SAM; F, LPS + SAM + AOAA; G, LPS + AOAA. n = 3. **p < 0.01 vs Con, ***p < 0.001 vs Con; ###p < 0.001 vs LPS

SAM treatment alone increased the intracellular Rhodamine 123 content of astrocytes (p < 0.01), while the AOAA group had a lower intracellular Rhodamine 123 content than the control group (p < 0.01) (Fig. 8). Besides, the LPS, LPS + AOAA + SAM, and LPS + AOAA groups showed significantly reduced intracellular Rhodamine 123 content (p < 0.001). However, there was no significant difference between the LPS + SAM and control groups. Compared with the LPS group, the LPS + SAM group had an increased intracellular Rhodamine 123 content (p < 0.001), while the intracellular Rhodamine 123 content of the LPS + AOAA and LPS + SAM + AOAA groups did not change significantly (Fig. 8).

**Fig. 9:**
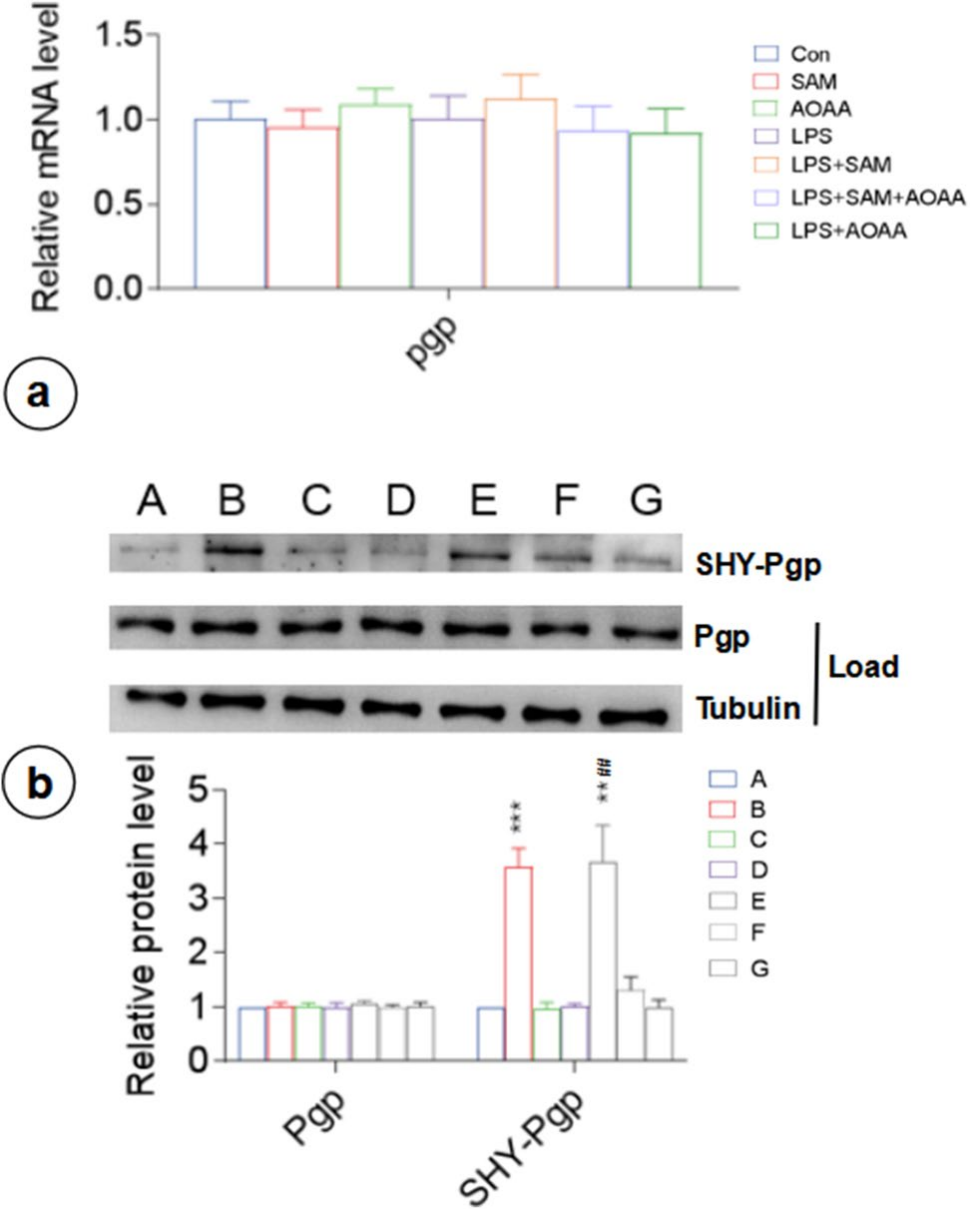
Endogenous H_2_S affects P-gp sulfation in astrocytes without affecting its expression at mRNA and protein levels. **a** RT-PCR of P-gp-encoding gene expression in astrocytes. **b** Biotin switch (S-sulfhydration) analysis to detect the level of P-gp sulfation in astrocytes. A, Con; B, SAM; C, AOAA; D, LPS; E, LPS + SAM; F, LPS + SAM + AOAA; G, LPS + AOAA. n = 3. **p < 0.01 vs Con, ***p < 0.001 vs Con; ##p < 0.001 vs LPS

Similar to the effects of exogenous sodium hydrosulfide, the alteration of CBS activity did not change P-gp expression at mRNA and protein levels (p > 0.05) (Fig. 9a, b). S-sulfhydration assay showed that the SAM and LPS + SAM groups had significantly higher levels of S-sulfhydrated P-gp than the control group (Fig. 9b). In contrast, there were no significant changes in the level of S-sulfhydrated P-gp in the other groups. The results indicated that endogenous CBS also regulated the H_2_S-mediated S-sulfhydration and altered the transport of P-gp, eventually affecting the secretion of inflammatory factors.

## Discussion

The inflammatory activation of astrocytes is an important pathological mechanism of neurodegenerative diseases, as it triggers the biosynthesis and secretion of toxic substances, including inflammatory mediators, which cause an imbalance in the nervous system microenvironment. Here, we showed that the mRNA expression levels of inflammatory factors (*Il1b*, *Il6*, and *tnfa*) and their extracellular levels in the culture supernatant increased significantly following LPS-induced inflammatory activation of astrocytes. Both exogenous and endogenous H_2_S significantly inhibited the inflammatory activation of astrocytes and reduced the production of inflammatory factors. More importantly, we found for the first time that H_2_S also regulated the secretion of inflammatory factors from astrocytes via S-sulfhydration of P-gp.

The LPS-induced neuroinflammatory response is often employed to simulate the inflammatory process in neurodegenerative diseases [27]. LPS, an inducer of inflammation, triggers the overexpression of inflammatory mediators (e.g., IL-1β, IL-6, and TNF-α) mainly by inducing the activation of the NF-κB pathway, resulting in a series of cellular and tissue injuries [28–30]. In this study, both exogenous and endogenous (CBS activator) increases in H_2_S levels significantly inhibited LPS-induced inflammatory response and the expression of proinflammatory factors in astrocytes. This finding is consistent with previous studies, which showed that inflammatory stimulation reduces CBS activity and H_2_S biosynthesis in glial cells, thereby enhancing the inflammatory response, while both endogenous and exogenous H_2_S can suppress the inflammatory response [18, 31]. As previously reported, NF-κB activation requires two key steps [25]: The transfer from the cytoplasm to the nucleus, and the post-transcriptional modification, such as phosphorylation. Therefore, nuclear translocation of P65, or phosphorylation of P65, is often used to assess the activation of NF-κB. Since it has been reported that NF-κB can be S-sulfhydrated [32], to explore whether H_2_S affects the nuclear translocation of NF-κB, we used the nuclear translocation of P65 and assessed NF-κB activation. The results show that H_2_S reduces NF-κB 65 entry into the nucleus, inhibiting NF-κB pathway activation.

Subsequent analyses uncovered that H_2_S not only affected the expression of proinflammatory factors in reactive astrocytes but also participated in the regulation of the secretion of proinflammatory factors. An increasing amount of data demonstrated that astrocytes are secretory cells of the nervous system [33]. Under normal circumstances, astrocytes generate and secrete neuroactive substances to maintain neural development and homeostasis of the nervous system microenvironment. Upon exposure to abnormal stimulation (e.g., LPS) or in the case of neurodegenerative diseases, activated astrocytes proliferate extensively and release various neurotoxins, including proinflammatory factors, which cause persistent damage to surrounding cells, promoting the pathological development of the disease. Neuroinflammatory responses can be alleviated to achieve neuroprotective effects by modulating the secretion of inflammatory mediators from reactive astrocytes [34]. In this study, the comparative analysis of the intracellular and extracellular (culture supernatant) levels of proinflammatory factors in reactive astrocytes showed that H_2_S reduced the secretion of proinflammatory factors in a concentration-dependent manner, which helped to contain the spread of inflammatory responses. The results indicated that H_2_S could regulate the secretion of proinflammatory factors from reactive astrocytes.

Under pathological conditions, astrocytes secrete proinflammatory cytokines (including IL-1β, IL-6, and TNF-α) that affect the function and microenvironment of surrounding cells, but the specific molecular mechanism remains unclear. Our results showed that the exposure of astrocytes to LPS significantly enhanced the transport of P-gp, and that treatment with the P-gp inhibitor verapamil inhibited the transport of P-gp; meanwhile, LPS-induced secretion of proinflammatory factors (IL-1β, IL-6, and TNF-α) was significantly suppressed. This is the first study reporting that P-gp may be an important component mediating the secretion of proinflammatory factors from reactive astrocytes.

P-gp, as an efflux pump of foreign molecules, has been widely studied for its roles in drug-resistant epilepsy and tumors, as well as maintenance of the blood–brain barrier [35–37]. However, there is increasing evidence to suggest the immunomodulatory effects of P-gp [9, 10, 38]. Previous animal studies have shown that the P-gp inhibitor verapamil reduces the serum levels of inflammatory mediators (e.g., TNF-α and IL-6) and improves the survival rate of mice upon LPS-induced septic shock [39]. It has been found that P-gp overexpression during inflammation promotes LPS-induced secretion of inflammatory mediators from nasal epithelial cells, leading to the onset and development of chronic rhinitis [40]. The role of P-gp in neuroinflammation has also received increasing attention, as it may promote neuroinflammation by participating in the secretion of inflammatory mediators [41]. A previous study on MS found that the enhanced transport of P-gp can increase the secretion of CCL2 from reactive astrocytes and induce the migration of monocytes, aggravating the neuroinflammatory responses [42].

Our results showed that exogenous H_2_S supplementation significantly inhibited the LPS-induced enhancement of P-gp-mediated transport, whereby the secretion of inflammatory factors (IL-1β, IL-6, and TNF-α) was reduced gradually with the increasing concentration of sodium hydrosulfide. Additionally, it has also been confirmed via endogenous activation or inhibition of H_2_S biosynthesis that H_2_S can affect the secretion of IL-1β and TNF-α by regulating their transport by P-gp. Similar to the inhibition of P-gp activity by verapamil, the inhibition of P-gp by NaHS and SAM led to the reduction of LPS-induced secretion of inflammatory factors. The accumulation of cytoplasmic inflammatory factors caused by the decrease in the secretion of inflammatory factors might reduce the transcription of nuclear genes. However, for different cells under different stimulation conditions, mRNA expression of inflammatory factors is affected to different extents in different reports [11, 43]. Interestingly, cytoplasmic inflammatory factor levels, increased due to LPS stimulation, were not significantly affected after the inhibition of P-gp activity. The synthesis and degradation of cytoplasmic inflammatory factors probably delayed the changes in their levels, and in the future, more time points need to be investigated to explore the relationship between time and inflammatory gene expression, as well as time and cytoplasmic inflammatory factor levels.

H_2_S may regulate P-gp transport by affecting protein expression and structural modifications. We found that H_2_S did not significantly alter P-gp expression at mRNA and protein levels but allowed S-sulfhydration of P-gp in a concentration-dependent manner, thereby leading to the reduced capacity of reactive astrocytes to secrete proinflammatory factors. It has been previously shown that S-sulfhydration is the most important mechanism by which H_2_S alters protein function [17]. S-sulfhydration refers to the H_2_S-mediated posttranslational modification of cysteine residues that results in functional changes in various proteins and enzymes. The research on S-sulfhydration is still in its infancy, but accumulating evidence suggests that S-sulfhydration may be the main mechanism underlying the neuroprotective properties of H_2_S, as many S-sulfhydrated proteins are key regulators in various neurodegenerative diseases [44, 45]. H_2_S levels and protein S-sulfhydration in the body decrease with age, rendering proteins susceptible to oxidative damage [46]. S-sulfhydration occurs in various neurological diseases, e.g., AD, PD, and MS [47, 48]. Supplementation with H_2_S or endogenous activation of CBS to increase the levels of relevant S-sulfhydrated proteins can improve disease progression. For example, reduced levels of H_2_S and protein S-sulfhydration in patients with AD led to the hyperphosphorylation of the microtubule-associated protein Tau, whereas H_2_S supplementation partially reversed the pathological changes in AD, as well as the decline of cognitive and behavioral functions [49].

## Conclusions

**Fig. 10:**
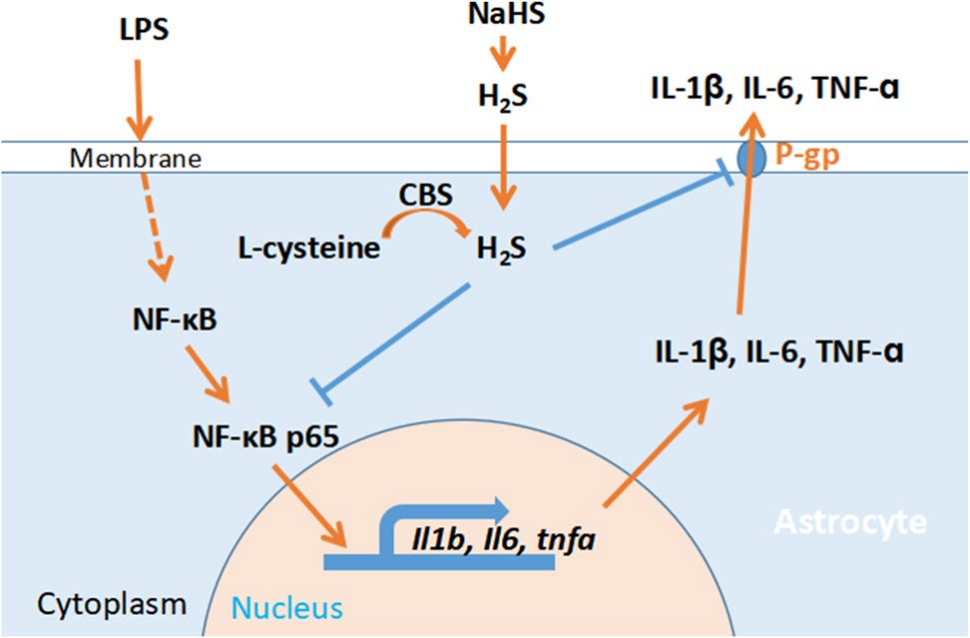
Schematic representation of the results

Our study indicates that H_2_S reduces LPS-induced activation of astrocytes and exerts anti-neuroinflammatory effects via multiple pathways (Fig. 10). On the one hand, H_2_S downregulates the expression and biosynthesis of proinflammatory factors by inhibiting the activation of the NF-κB pathway. On the other hand, H_2_S reduces the secretion of inflammatory factors by inhibiting P-gp-mediated transport via S-sulfhydration. Our study has provided new ideas and directions for studying mechanisms underlying the anti-inflammatory and neuroprotective effects of H_2_S. Our findings encourage further in vivo studies on the roles of H_2_S in inflammation-associated neurodegenerative diseases to explore new therapeutic targets and strategies for these neurological diseases.

## Supplementary Information

Below is the link to the electronic supplementary material.

Supplementary file1 (TIF 3600 kb) Primary astrocyte culture and identification. Red fluorescence signal represents GFAP positivity, blue fluorescence signal represents the nucleus, and a positive rate >95% indicates that cell purity is >95%

Supplementary file2 (TIF 818 kb) LPS induces inflammatory activation of primary astrocytes. LPS (1 μg/mL) induced increased *GFAP* mRNA expression in primary astrocytes. **p<0.01 vs control (Con)

Supplementary file3 (TIF 2636 kb) a. Cell viability of astrocytes after administration of verapamil (P-gp inhibitor, 70 µM). There was no significant difference between the groups. b. Cell viability of astrocytes after administration of H_2_S donor sodium hydrosulfide (NaHS, 50, 100, and 300 µM). There was no significant difference between the groups. c. Cell viability of astrocytes after administration of the CBS activator S-adenosyl-L-methionine (SAM, 0.1 mmol/L) and (or) the CBS inhibitor aminooxyacetic acid (AOAA, Sigma-Aldrich, 1 mmol/L). There was no significant difference between the groups

Supplementary file4 (TIF 2620 kb)

Supplementary file5 (TIF 2806 kb)

## Data Availability

All data relevant to the study are included in the article or uploaded as supplementary information.
