# Supplementary figures and images for: Hydrogen Sulfide Attenuates Lipopolysaccharide-Induced Inflammation via the P-glycoprotein and NF-κB Pathway in Astrocytes
Source: Neurochem Res. 2022 Dec 8;48(5):1424–37. doi: 10.1007/s11064-022-03840-5 (PMC10066098; doi:10.1007/s11064-022-03840-5)

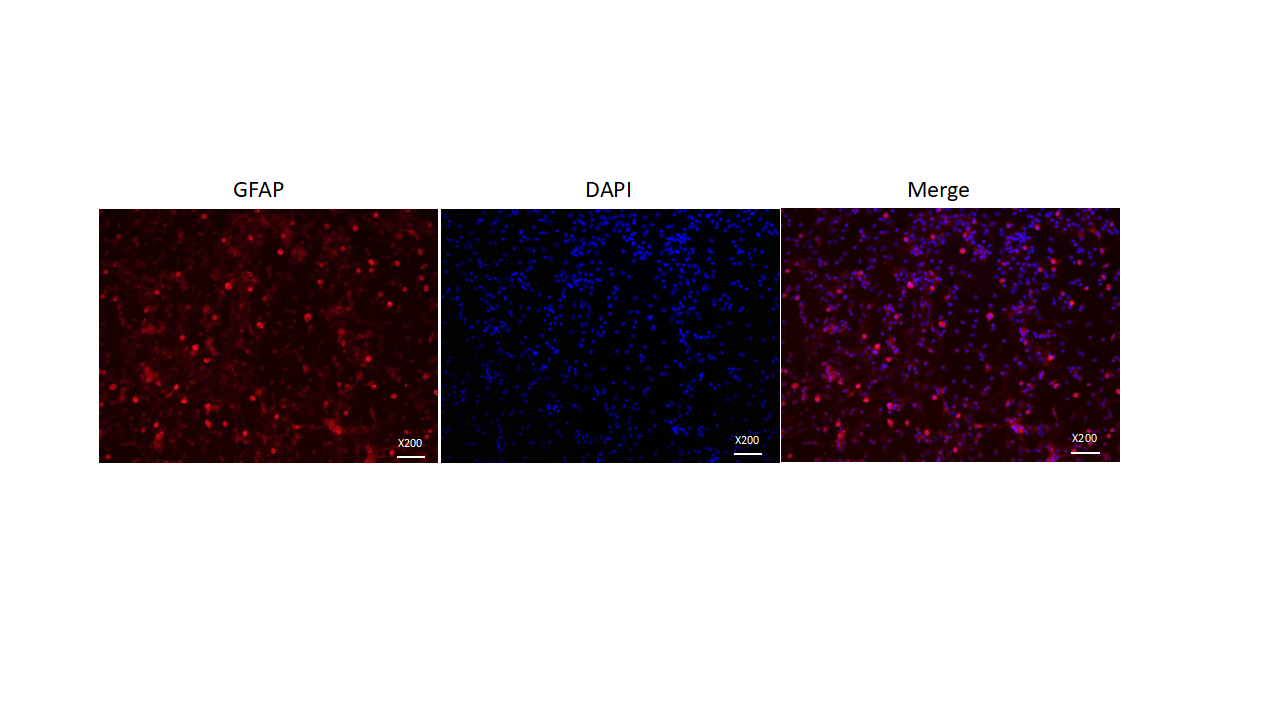

Supplement: Supplementary file 1 — Supplementary file1 (TIF 3600 kb) Primary astrocyte culture and identification. Red fluorescence signal represents GFAP positivity, blue fluorescence signal represents the nucleus, and a positive rate >95% indicates that cell purity is >95% [file 11064_2022_3840_MOESM1_ESM.tif]

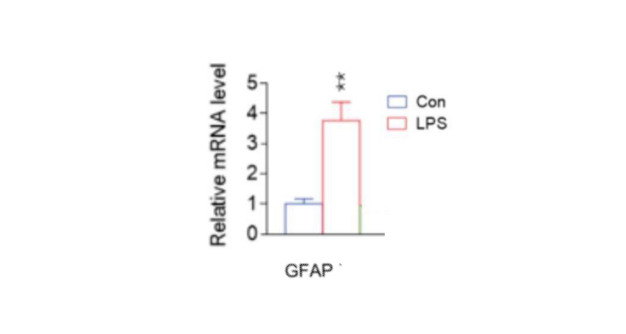

Supplement: Supplementary file 2 — Supplementary file2 (TIF 818 kb) LPS induces inflammatory activation of primary astrocytes. LPS (1 μg/mL) induced increased GFAP mRNA expression in primary astrocytes. **p<0.01 vs control (Con) [file 11064_2022_3840_MOESM2_ESM.tif]

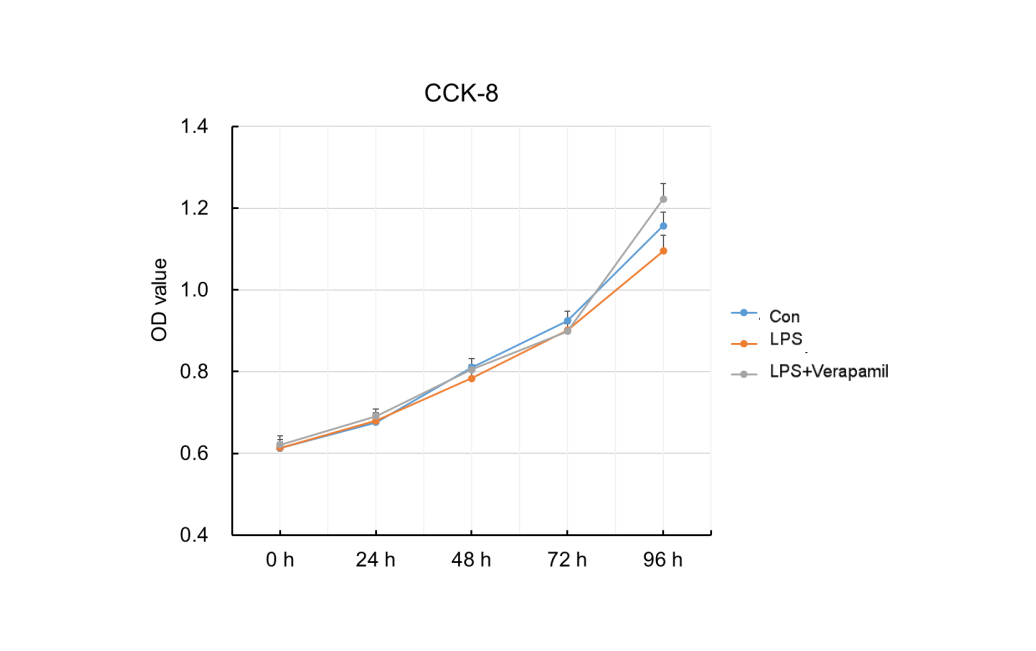

Supplement: Supplementary file 3 — Supplementary file3 (TIF 2636 kb) a. Cell viability of astrocytes after administration of verapamil (P-gp inhibitor, 70 µM). There was no significant difference between the groups. b. Cell viability of astrocytes after administration of H2S donor sodium hydrosulfide (NaHS, 50, 100, and 300 µM). There was no significant difference between the groups. c. Cell viability of astrocytes after administration of the CBS activator S-adenosyl-L-methionine (SAM, 0.1 mmol/L) and (or) the CBS inhibitor aminooxyacetic acid (AOAA, Sigma-Aldrich, 1 mmol/L). There was no significant difference between the groups [file 11064_2022_3840_MOESM3_ESM.tif]

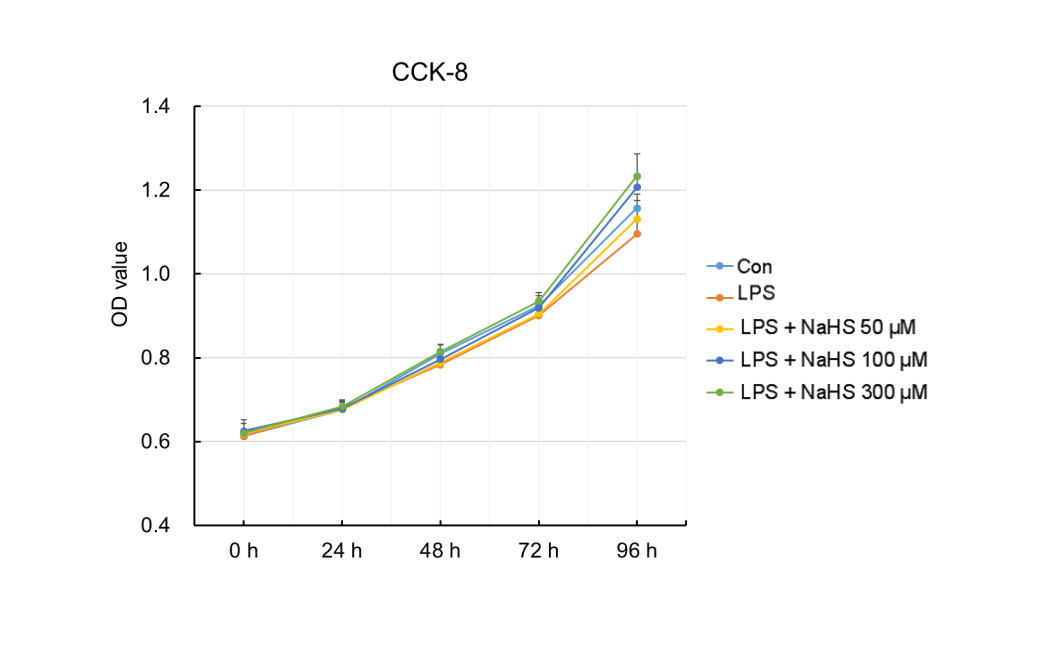

Supplement: Supplementary file 4 — Supplementary file4 (TIF 2620 kb) [file 11064_2022_3840_MOESM4_ESM.tif]

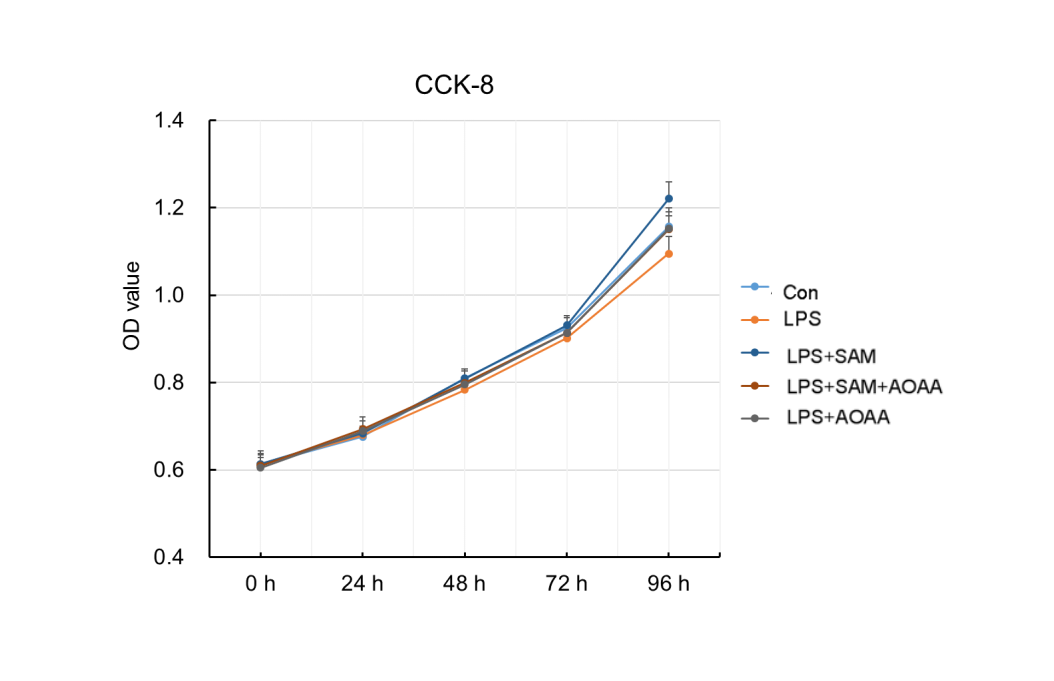

Supplement: Supplementary file 5 — Supplementary file5 (TIF 2806 kb) [file 11064_2022_3840_MOESM5_ESM.tif]
